# Triple-Negative Breast Cancer: A Retrospective Study of 23 Cases in Morocco

**DOI:** 10.7759/cureus.80756

**Published:** 2025-03-18

**Authors:** Leila Abdallaoui Maane, Nabil Ismaili, Abderrahim Siati, Mohamed Fareh, Adil Elghanmi, Amal Bouziyane, Mohamed Dehayni

**Affiliations:** 1 Obstetrics and Gynecology, Cheikh Khalifa International University Hospital, Mohammed VI University of Health Sciences (UM6SS), Casablanca, MAR; 2 Medical Oncology, Cheikh Khalifa International University Hospital, Mohammed VI University of Health Sciences (UM6SS), Casablanca, MAR

**Keywords:** breast cancer epidemiology, breast cancer subtypes, chemotherapy, immunohistochemistry, triple-negative breast cancer

## Abstract

Introduction

Triple-negative breast cancer (TNBC) is characterized by the absence of estrogen and progesterone hormone receptor expression in tumor cells, and the lack of human epidermal growth factor receptor 2 (HER2) growth factor expression on immunohistochemistry. It is considered an aggressive subtype with limited treatment options, based mainly on chemotherapy, surgery, and radiotherapy.

Materials and methods

This retrospective study examines the epidemiological, clinical, pathological, and therapeutic characteristics of TNBC. It is based on a series of 23 cases collected from the Medical Oncology Department of the Cheikh Khalifa Ibn Zaid Al Nahyan International University Hospital in Casablanca, Morocco, over a period of three years and six months. The study focuses on newly diagnosed patients with TNBC, with data collected from the medical records of patients who were diagnosed and treated during this period. Follow-up data were analyzed to assess disease progression and treatment response, but all cases included were diagnosed during the study period.

Results

Of the total 242 breast cancer cases, 12.4% (30 cases) were diagnosed with TNBC. Due to missing data, only 23 cases were included in the final analysis. The average age of patients was 51.35 years, and 26.09% (six patients) had a family history of breast cancer. Invasive ductal carcinoma (IDC) was the most common histological type, accounting for 91.30% (21 cases) of cases, with a mean tumor size of 40.69 mm. Histoprognostic grades II and III were predominant, representing 30.43% (seven cases) and 52.17% (12 cases) of cases, respectively. Surgical treatments included conservative lumpectomy in 43.48% (10 patients), radical mastectomy according to the Patey technique in 26.09% (six patients), and no surgery for 30.43% (seven patients). Chemotherapy was administered to 69.6% (16 patients) of patients, with 39.1% (nine patients) receiving adjuvant therapy and 30.4% (seven patients) undergoing neoadjuvant treatment. Lymph node involvement was present in 63.64% (14 patients) of cases, with bone metastasis in 45.45% (10 patients) and lung metastasis in 27.27% (six patients). The disease course was considered unfavorable in 55% (13 patients) of cases, with disease progression observed in these patients. Specifically, 35% developed local recurrence, and 35% experienced metastatic progression. The average recurrence-free survival was 22.74 months post-diagnosis.

Conclusion

TNBC is a highly aggressive form of breast cancer, associated with a poor prognosis due to its high metastatic potential and frequent recurrence, particularly within the first two years after diagnosis. Current therapeutic options remain limited, highlighting the ongoing need for improved treatment strategies and early detection efforts.

## Introduction

Breast cancer is the most prevalent malignancy among women worldwide, accounting for 35.7% of cases, and remains the leading cause of cancer-related mortality in women [1]. It is estimated that approximately two million cases are diagnosed annually, resulting in 627,000 deaths [1]. In Morocco, breast cancer represents a significant public health concern due to its increasing incidence and its tendency to affect younger women [2,3]. The heterogeneity of breast cancer at clinical, histological, and molecular levels contributes to variations in tumor presentation, treatment response, and prognosis [3].

Triple-negative breast cancer (TNBC) is a distinct and aggressive subtype of breast cancer, characterized by the absence of estrogen receptor (ER), progesterone receptor (PR), and human epidermal growth factor receptor 2 (HER2) expression. Representing approximately 15%-20% of all breast cancers, TNBC lacks targeted therapies, such as endocrine therapy or HER2-targeted agents [4]. As a result, chemotherapy remains the mainstay of systemic treatment, alongside surgery and radiotherapy, which contributes to its poor prognosis [5]. TNBC is associated with a higher risk of early recurrence, distant metastases, and lower survival rates, particularly within the first five years following diagnosis [5].

Given the aggressive nature and limited treatment options for TNBC, this subtype has become a major focus of research. In this study, we present an analysis of 23 cases of TNBC, diagnosed and treated in a tertiary care hospital in Casablanca over a period of three and a half years, from 2018 to 2021. Through this case series and a comprehensive literature review, we aim to provide insights into the epidemiological, clinical, pathological, therapeutic, and prognostic characteristics of TNBC.

## Materials and methods

Study design

This study is a retrospective case series conducted at the Medical Oncology Department of Cheikh Khalifa International University Hospital in Casablanca, Morocco. It analyzes 23 cases of TNBC diagnosed and treated over a period of three and a half years (from 2018 to 2021). Data were collected from institutional electronic medical records, including the DXCare and LIMS systems, to ensure comprehensive retrieval of patient information. Ethical approval was obtained from the Ethics Committee of Cheikh Khalifa Ibn Zaid Al Nahyan International University Hospital, Casablanca, Morocco. Informed consent was waived due to the retrospective nature of the study, as the analysis was based solely on de-identified patient medical records in compliance with ethical standards for retrospective research.

Study objectives 

The primary objective of this study was to describe the epidemiological, clinical, pathological, therapeutic, and prognostic characteristics of TNBC. By evaluating patient demographics, tumor biology, treatment regimens, and disease outcomes, we aimed to identify potential patterns that could aid in optimizing TNBC management.

Inclusion and exclusion criteria 

The study included female patients diagnosed with TNBC, confirmed through immunohistochemistry. Only patients who received treatment at Cheikh Khalifa International University Hospital were considered eligible for inclusion. Male patients were excluded from the study, as were cases with incomplete histopathological data. Additionally, patients who had been treated elsewhere or had incomplete medical records that hindered proper analysis were not included in the final dataset.

Data collection 

Relevant clinical and pathological data were systematically extracted from electronic medical records. The demographic and clinical parameters recorded included patient age, menopausal status, parity, breastfeeding history, use of oral contraceptives, and family or personal history of breast cancer. Tumor characteristics, such as tumor size, histological type, Scarff-Bloom-Richardson (SBR) grade, Ki67 proliferation index, vascular invasion, lymph node involvement, and metastatic status, were also documented.

The diagnostic workup was reviewed to assess imaging findings obtained from mammography, ultrasound, computed tomography (CT) scans, and bone scintigraphy. Histopathological and biomarker analyses, including ER, PR, HER2, Ki67 index, and molecular profiling, were also recorded.

Regarding treatment modalities, we collected detailed data on the type of surgery performed, distinguishing between mastectomy and lumpectomy. Information on chemotherapy regimens was categorized based on whether treatment was administered in a neoadjuvant, adjuvant, or metastatic setting. Data on radiotherapy, including dosage and fractionation schedules, were also documented, along with any toxicities observed throughout the course of treatment.

Patient outcomes and follow-up data were meticulously analyzed to assess disease progression, recurrence patterns, metastasis sites, and overall survival. This enabled the evaluation of long-term prognosis and treatment effectiveness among the studied cases.

Statistical analysis

Data analysis was conducted using Microsoft Excel (Microsoft® Corp., Redmond, WA, USA) and IBM SPSS Statistics for Windows, Version 28 (Released 2021; IBM Corp., Armonk, NY, USA). Descriptive statistics were employed to summarize categorical variables as frequencies and percentages, while continuous variables were presented as means, medians, and standard deviations. To assess disease outcomes, Kaplan-Meier survival curves were generated to estimate progression-free survival (PFS). Additional statistical tests were applied, as appropriate, to explore associations between clinicopathological features and patient prognosis.

By providing a detailed account of the methodology used, this study ensures a robust analysis of TNBC cases, offering insights into disease presentation, management, and outcomes in our patient cohort.

## Results

Epidemiological data 

**Figure 1:**
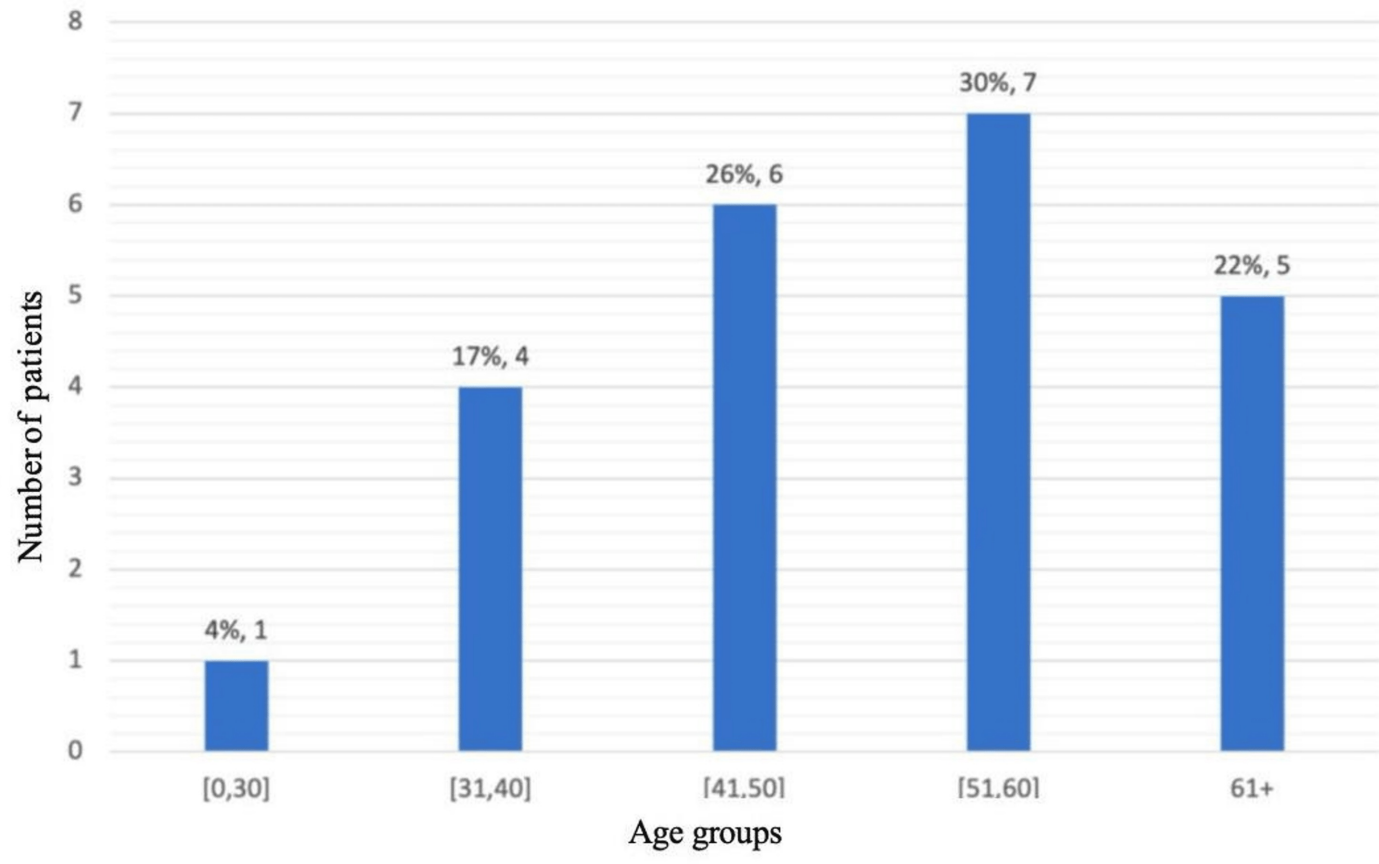
Distribution of patients by age group

Among the 242 breast cancer cases reviewed during the study period, 30 (12.4%) were diagnosed with TNBC. Due to missing data, only 23 cases were included in the final analysis. The mean age of the patients was 51.35 years (SD: 12.94; range: 27-76 years), with five patients (21.74%) under 40 years old (Figure 1).

A family history of breast cancer was reported in six cases (26.09%), though BRCA1/2 (breast cancer 1/2) genetic testing was not routinely performed. Regarding reproductive factors, 12 patients (52.17%) were multiparous, with 21 cases (91.67%) having a history of breastfeeding. Oral contraceptive use was recorded in eight cases (34.78%).

Clinical and tumor characteristics

**Figure 2:**
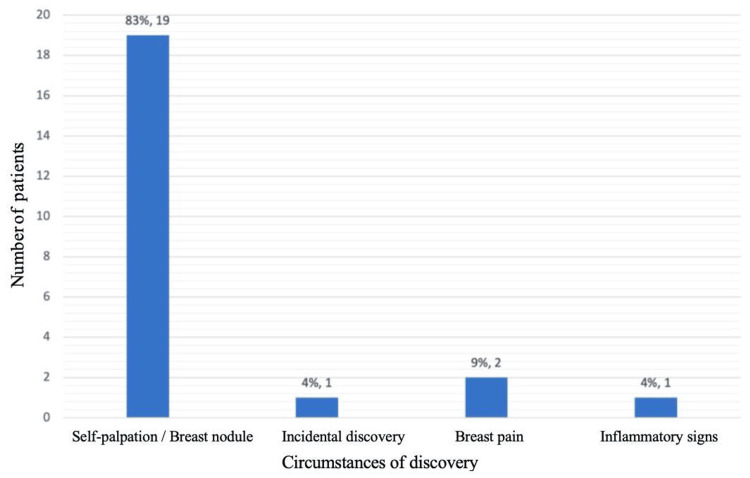
Representation of the circumstances of cancer discovery

The most common presenting symptom was a palpable breast mass, reported in 19 patients (82.61%), followed by breast pain in two patients (8.69%) and inflammatory changes in one patient (4.35%). Only one case (4.35%) was diagnosed incidentally through imaging (Figure 2).

**Figure 3:**
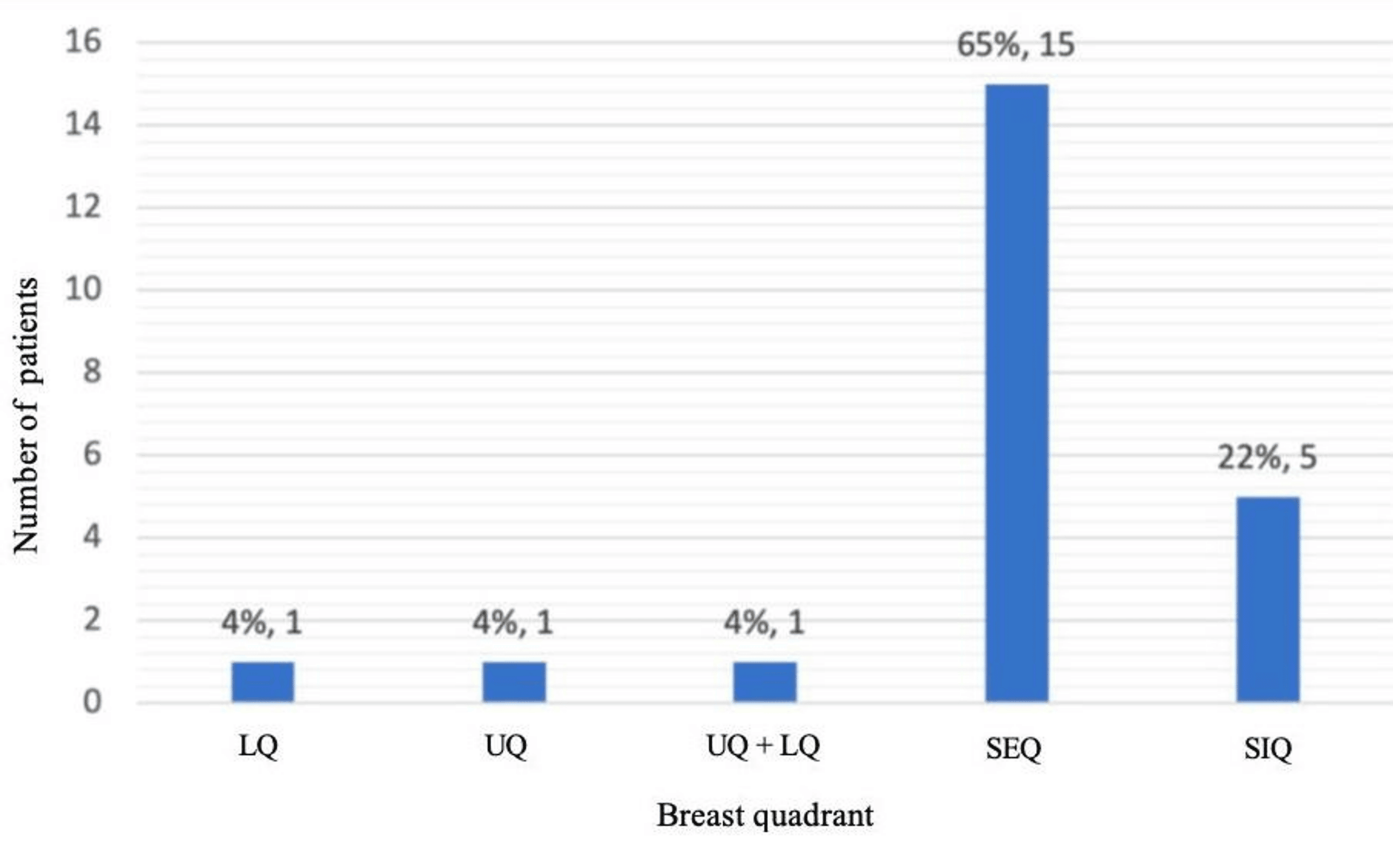
Distribution according to the location of the nodule LQ: Lower quadrant; UQ: Upper quadrant; UQ + LQ: Upper and lower quadrants; SEQ: Supero-external quadrant; SIQ: Supero-internal quadrant

Tumors were more frequently located in the upper outer quadrant (15 patients, or 65.22%), with an almost equal distribution between the right (11 patients, or 47.83%) and left breast (12 patients, or 52.17%) (Figure 3).

**Figure 4:**
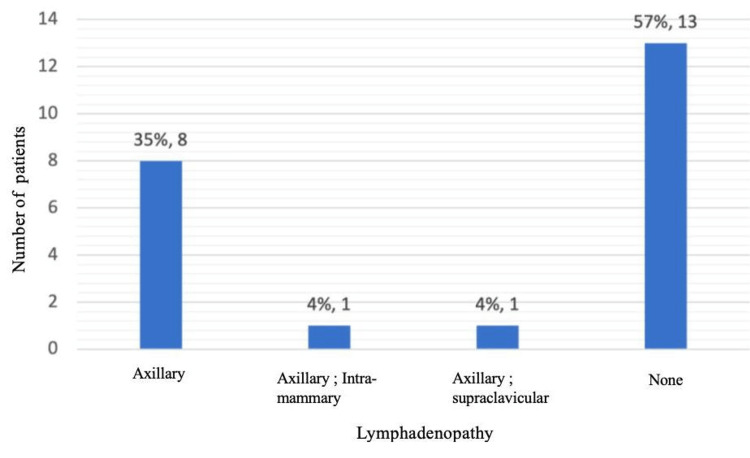
Distribution of patients according to the presence of lymphadenopathy

No bilateral cases were observed. Lymph node involvement was clinically detected in 10 patients (43.48%), primarily affecting the axillary nodes (Figure 4).

Histopathological and molecular features

The predominant histological type was invasive ductal carcinoma (IDC) (21 cases, or 91.30%), followed by invasive lobular carcinoma and medullary carcinoma (one case each). Tumor size ranged from 14 mm to 120 mm, with a median size of 30 mm. The majority of tumors (19 patients, or 82.61%) were ≥2 cm. SBR grade III tumors were predominant (12 cases, or 52.17%), followed by grade II (seven cases, or 30.43%). The Ki67 proliferation index was available for 19 cases, with all tumors exhibiting a high proliferation rate (>20%). Vascular invasion was reported in seven cases (28.57%), and lymph node invasion was confirmed histologically in seven cases (30.43%) of surgical specimens.

Diagnostic imaging findings 

Mammography results showed that three cases (13.04%) were classified as Breast Imaging-Reporting and Data System (BI-RADS-4) (suspicious abnormality), while 13 cases (56.52%) were classified as BI-RADS-5 (highly suggestive of malignancy). CT scans were performed in 21 cases (91.30%), detecting distant metastases in nine cases (38.09%). Bone scintigraphy was performed in 17 cases (73.91%), revealing metastases in four cases (17.65%). Liver metastases were detected in three cases (13.04%), bone metastases in three cases (13.04%), pulmonary metastases in two cases (8.69%), and peritoneal carcinomatosis in one case (4.35%).

Treatment and response 

Lumpectomy (breast-conserving surgery) was performed in 10 cases (43.48%), while mastectomy was performed in six cases (26.09%). A total of seven patients (30.43%) did not undergo surgery due to metastatic disease or other contraindications. Chemotherapy was administered to 21 patients (91.30%), with eight (33.33%) receiving neoadjuvant chemotherapy, 10 (42.86%) receiving adjuvant chemotherapy, and five (23.81%) receiving both. The most frequently used protocols included anthracyclines and taxanes, with a mean of 7.68 chemotherapy cycles (range: 4-12 cycles). Adjuvant radiotherapy was delivered to 14 patients (60.87%), with a median dose of 49 Gy over 23.28 sessions. Common toxicities included radiodermatitis (eight patients, or 35.71%) and oral mucositis (seven patients, or 28.57%).

Survival and prognostic factors 

Disease progression occurred in 12 patients (55%), with eight patients (35%) developing local recurrence and eight patients (35%) experiencing metastatic progression. The most common metastatic sites were lymph nodes (14 patients, or 63.64%), bone (10 patients, or 45.45%), lung (six patients, or 27.27%), brain (four patients, or 18.18%), and liver (four patients, or 18.18%). Prognostic analysis indicated that patients with tumors ≤2 cm had better outcomes, achieving 100% disease control. In contrast, SBR grade III tumors were associated with a worse prognosis, with a progression rate of 55.56%. Additionally, patients with disease progression had a higher Ki67 index (58.5% vs. 51.25%).

**Figure 5:**
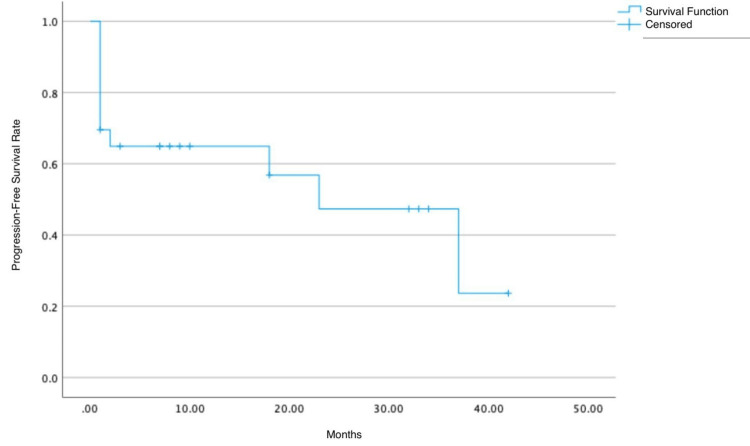
Estimation of the progression-free survival curve

A PFS curve was generated using the Kaplan-Meier method to account for censored data, including patients lost to follow-up or with interrupted follow-up. This curve represents the probability of relapse-free survival over time, ranging from 0 to 42 months (Figure 5).

Of the 23 patients in total, 11 experienced relapse. The remaining 12 patients - nine who had not relapsed by the time of follow-up and three who were lost to follow-up - were censored. The estimated average PFS was 22.74 months, with a median PFS of 23 months. The probability of survival at one year was 69.6%, at two years was 47.3%, and at three years was 23.7%.

**Figure 6:**
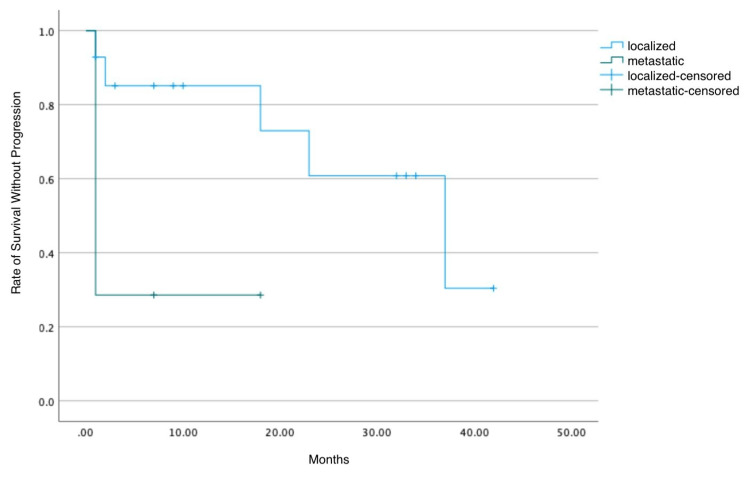
Estimation of the progression-free survival curve (metastatic vs. localized)

When comparing patients with localized versus metastatic cancer, the Kaplan-Meier method revealed significant differences (Figure 6).

Among the 21 patients with available data, 14 were diagnosed with localized cancer (five relapses and nine censored), and seven were diagnosed with metastatic cancer (five relapses and two censored). For patients with localized cancer, the average PFS was 29.23 months, with a median survival of 37 months. In contrast, those with metastatic cancer had an average PFS of only 5.86 months and a median survival of just one month. For patients with localized cancer, the one-year survival probability was 85.1%, 60.8% at two years, and 30.4% at three years. For patients with metastatic cancer, the survival probability was much lower, dropping to 28.6% within the first few months of follow-up.

## Discussion

TNBC is an aggressive subtype representing 12.4% of all breast cancer cases in our study, aligning with global estimates of 10%-20% [1]. The mean age at diagnosis was 51.35 years, with 21.74% of patients under 40 years old, confirming that TNBC disproportionately affects younger women compared to hormone receptor-positive breast cancers [1,2]. A family history of breast cancer was reported in 26.09% of cases, though BRCA1/2 mutation testing was not performed. Previous studies suggest that 15%-45% of TNBC cases harbor BRCA1 mutations, emphasizing the importance of genetic screening [3].

Most TNBC cases presented with a palpable breast mass (82.61%), consistent with literature indicating that TNBC is rarely detected incidentally [4]. Lymph node involvement was found in 43.48% of cases, reflecting its aggressive biology. Histologically, IDC (91.30%) was the predominant subtype, and 52.17% of tumors were high-grade (SBR III), reinforcing TNBC’s poor differentiation [5]. The Ki67 proliferation index was high (>20%) in all cases, a known predictor of poor prognosis [6].

Mammography classified 56.52% of TNBC tumors as BI-RADS-5, reflecting the well-documented aggressive radiological features of TNBC [7]. Metastatic workup revealed that 34.78% of patients had metastatic disease at diagnosis, with the most common sites being lymph nodes (63.64%), bone (45.45%), lungs (27.27%), liver (18.18%), and brain (18.18%), consistent with TNBC’s known hematogenous spread pattern [8].

TNBC remains a challenging subtype due to its aggressive nature and limited targeted therapy options. Given the lack of targeted therapies, chemotherapy remains the cornerstone of TNBC treatment. In our cohort, 91.30% of patients received chemotherapy, with 33.33% undergoing neoadjuvant therapy. The most commonly used regimens included anthracyclines and taxanes, which are standard for TNBC management [9]. Surgical management varied, with 43.48% undergoing lumpectomy, 26.09% undergoing mastectomy, and 30.43% not eligible for surgery due to advanced disease. Adjuvant radiotherapy was administered to 60.87% of cases, with a median dose of 49 Gy, consistent with clinical guidelines [10].

Disease progression was observed in 55% of cases, with 35% developing local recurrence and 35% progressing to metastatic disease. Key prognostic factors included tumor size, as patients with tumors ≤2 cm had better outcomes, highlighting the importance of early detection [11]. Histological grade played a significant role, with SBR grade III tumors exhibiting a 55.56% progression rate, confirming its association with a worse prognosis [12]. Additionally, a higher Ki67 index (>50%) correlated with poorer outcomes, reinforcing its prognostic value in TNBC [13].

Our findings align with global TNBC data, confirming its high-grade pathology, early metastasis, and poor prognosis [14]. However, the lack of BRCA genetic testing in our cohort highlights a gap in screening strategies, as BRCA-mutated TNBC patients may benefit from PARP (poly(ADP-ribose) polymerase) inhibitors [15]. Additionally, our progression rate (55%) is slightly higher than some Western cohorts (40%-50%), potentially due to later-stage diagnosis and limited access to immunotherapy. Immunotherapy has shown promise in TNBC, particularly PD-L1 (programmed death-ligand 1) inhibitors such as atezolizumab and pembrolizumab, which significantly improve survival in PD-L1-positive TNBC [16].

Recent advancements in TNBC treatment have focused on immunotherapy and targeted therapies. Immunotherapy with immune checkpoint inhibitors (ICIs), particularly PD-L1 inhibitors like atezolizumab and pembrolizumab, has demonstrated improved PFS and overall survival in PD-L1-positive TNBC [16]. Pembrolizumab, in combination with chemotherapy, is now FDA-approved for metastatic TNBC with PD-L1 expression. Furthermore, PARP inhibitors, such as olaparib and talazoparib, have shown efficacy in patients with germline BRCA mutations, offering a targeted treatment option for this TNBC subgroup [17,18]. The combination of ICIs with PARP inhibitors is currently under investigation and may provide synergistic benefits by enhancing tumor immunogenicity. Additionally, antibody-drug conjugates (ADCs), such as sacituzumab govitecan, have emerged as a promising treatment option for relapsed or refractory TNBC, with significant clinical benefits in heavily pretreated patients [19].

Despite the challenges associated with TNBC, advancements in genomic profiling and immunotherapy offer new therapeutic avenues. The identification of molecular subtypes within TNBC, such as basal-like and mesenchymal phenotypes, could further refine treatment strategies. Moreover, incorporating biomarkers like PD-L1 expression, tumor-infiltrating lymphocytes (TILs), and the Ki67 index into clinical decision-making may help identify patients who are more likely to benefit from immunotherapy and combination regimens [16].

 Limitations and future directions

This study has several limitations. The small sample size (23 cases) restricts the generalizability of our findings, making it difficult to draw definitive conclusions applicable to broader populations. Additionally, the lack of BRCA1/2 genetic testing prevents the establishment of a correlation between hereditary TNBC and disease outcomes, limiting insights into potential genetic predispositions. Another major limitation is the absence of novel targeted therapies, such as PARP inhibitors and ICIs, which have demonstrated promising results in recent trials [9]. The exclusion of these therapies may have impacted survival outcomes and the overall treatment efficacy observed in this study.

Despite these limitations, the study also has notable strengths. It provides valuable regional data on TNBC in Morocco, filling a gap in the literature concerning North African populations. The detailed clinicopathological data, including tumor characteristics, treatment modalities, and outcomes, offer a robust understanding of TNBC behavior in this cohort. Furthermore, the study's methodological rigor, with systematic data collection from electronic medical records and thorough statistical analysis, enhances the validity of its findings. The evaluation of treatment outcomes, such as PFS, provides critical insights into the effectiveness of current therapeutic approaches in the region.

Future research should prioritize larger, multicenter cohorts to enhance statistical power and better represent the diverse clinical presentations of TNBC. Incorporating genetic profiling will provide a more comprehensive understanding of the molecular landscape of TNBC, allowing for personalized therapeutic approaches. Furthermore, evaluating the impact of immunotherapy on TNBC survival is essential, particularly in resource-limited settings where access to these treatments remains a challenge. Expanding treatment strategies and integrating novel targeted therapies may significantly improve patient outcomes in the future.

## Conclusions

TNBC remains an aggressive breast cancer subtype characterized by rapid progression, early metastases, and limited treatment options. Our study highlights that TNBC predominantly affects middle-aged women, with a significant proportion of cases occurring in those under 40 years old. The high-grade, large tumors at diagnosis underscore the need for improved early detection strategies to enhance patient outcomes. Despite standard multimodal treatment, disease progression and distant metastases remain common, emphasizing the need for more effective therapies.

Optimizing treatment through the integration of emerging therapies, such as immunotherapy and PARP inhibitors, holds promise for improving survival, especially in patients with BRCA mutations. Expanding access to genetic testing and enhancing healthcare provider training in resource-limited settings are critical to guiding personalized treatment. Future research should focus on identifying novel biomarkers, refining therapeutic strategies, and improving early diagnosis to reduce TNBC-related mortality and improve patient prognosis.
